# Smoking affects symptom improvement in schizophrenia: a prospective longitudinal study of male patients with first-episode schizophrenia

**DOI:** 10.1038/s41537-024-00449-1

**Published:** 2024-03-15

**Authors:** Xishu Mu, Wenjing Wu, Sisi Wang, Xiuru Su, Hengyong Guan, Xiaoni Guan, Xiaobing Lu, Zezhi Li

**Affiliations:** 1 Hebei Province Veterans Hospital, Baoding, China; 2 Qingdao Mental Health Center, Qingdao, China; 3 Peking University HuiLongGuan Clinical Medical School, Beijing HuiLongGuan Hospital, Beijing, China; 4 Department of Nutritional and Metabolic Psychiatry, The Affiliated Brain Hospital of Guangzhou Medical University, Guangzhou, China; 5 Guangdong Engineering Technology Research Center for Translational Medicine of Mental Disorders, Guangzhou, China; 6 Key Laboratory of Neurogenetics and Channelopathies of Guangdong Province and the Ministry of Education of China, Guangzhou Medical University, Guangzhou, China

**Keywords:** Schizophrenia, Psychiatric disorders

## Abstract

Patients with schizophrenia (SCZ) smoke up to three times more than general people. However, there are conflicting results regarding the relationship between tobacco smoke and clinical symptom severity in SCZ. The aim of this study was to assess the impact of smoking on clinical symptoms after antipsychotic treatment in a 12-week cohort study after controlling for confounding factors. One hundred and forty-five male patients with drug-naïve first-episode (DNFE) SCZ received antipsychotic monotherapy for 12 weeks. Symptom severity was assessed at baseline and at week 12 by the Positive and Negative Syndrome Scale (PANSS). We found no differences in clinical symptoms among male smokers with SCZ compared with male nonsmokers. However, male smokers showed greater improvement in negative symptoms after 12 weeks of treatment, controlling for age, years of education, onset age, and baseline body mass index (BMI). Our study showed that after 12 weeks of treatment with antipsychotics, male smokers showed greater improvement in negative symptoms than male nonsmokers.

## Introduction

Schizophrenia (SCZ) is a chronic and severe mental disorder that affects 1% of the world’s population. People with schizophrenia experience hallucinations, and disorganized thinking, and are prone to false and paranoid beliefs^1^. The symptoms of SCZ are classified as positive symptoms and negative symptoms.

The negative symptoms are defined as a reduction or lack of normal behaviors associated with normal functioning, such as social functioning, motivation, and personal expression. Negative symptoms can be primary symptoms inherent to the underlying pathophysiology of SCZ or secondary symptoms related to psychiatric or medical comorbidities, or adverse treatment effects^2^. The negative symptom domain consists of 5 key constructs: blunted affect, alogia (reduction in the number of words spoken), avolition (reduced goal-directed activity due to decreased motivation), asociality, and anhedonia (reduced experience of pleasure)^3,4^. Negative symptoms are one of the core symptoms of SCZ and are strongly associated with long-term morbidity and poor functional outcomes in patients^5,6^. While positive symptoms can often be effectively improved with available antipsychotic medications, current treatment options for negative symptoms are relatively limited in effectiveness, and they remain an unmet medical need^7^. Interestingly, some studies have shown evidence of fewer negative symptoms in SCZ patients who smoke, which contributes to the self-treatment of negative symptoms^8^.

Patients with SCZ have a high rate of smoking compared to the general population^9^. Previous studies have reported a smoking rate of ~60% in SCZ patients and a strong association between smoking and SCZ (OR = 6:0; 95% CI, 3.0–12.0)^10,11^. In particular, studies have shown that patients with SCZ are also more likely to be heavy smokers compared to the general population^12^. A study by our group showed that in China, the prevalence of heavy smoking was higher in SCZ patients (28.3%) than in the general population (14.5%)^13^.

Previous studies have shown that smoking can act as self-medication for clinical symptoms of SCZ through the neurobiological basis of nicotine^14^. A meta-analysis also reported that nicotine could enhance cognitive functioning, including motor abilities, attention and immediate memory^15^. Longitudinal studies have also reported that smoking may be an independent protective factor for the development of SCZ^16^. Nicotine is the main addictive component of cigarettes^17,18^, which has been reported to ameliorate the abnormalities in dopaminergic, glutamatergic, and GABAergic pathways^19^ and has been linked to the mechanism of symptom improvements.

However, the link between smoking and SCZ has been a complex issue. Some studies reported that smoking was associated with more severe negative symptoms in patients with psychiatric disorders^20^. A cross-sectional study of SCZ patients showed that mildly dependent smokers had more severe negative symptoms than nonsmokers^21^. A 4 to 16-year follow-up study including 14,248 healthy adolescents without any psychiatric disorder showed that those who smoked <9 cigarettes per day were 1.38 times more likely to be later hospitalized for SCZ and those who smoked >10 cigarettes per day were 2.28 times more likely to develop SCZ than nonsmokers (95% CI = 1:19–4.34)^22^. Another cohort study of 1,413,849 women and 233,879 men with first-episode SCZ showed a hazard ratio of 3.5 for women and 3.8 for men for smoking^23^, suggesting that cigarette smoking causes SCZ. Some confounding factors that were not controlled for, such as antipsychotic types, disease duration and demographic characteristics of patients could explain the discrepancy in a variety of studies.

To better understand the relationship between smoke and SCZ, in this study, we recruited male drug-naïve first-episode (DNFE) patients to exclude the potential effects of antipsychotic medication, duration of illness and sex. In clinical studies in the SCZ, patients with DNFE are particularly advantageous because the effects of confounding factors, including medication efficacy, duration of illness, and physical comorbidity, can be minimized^24^. In addition, to control for the effects of different antipsychotics, all patients in our study received antipsychotics as monotherapy. We hypothesized that smoking might alleviate negative symptoms in SCZ patients after treatment with antipsychotic monotherapy in male patients with DNFE SCZ. Because smoking is substantially more common among Chinese men than in women with SCZ^25^, as well as sex differences in smoking behaviors^26^, we recruited only male patients. Therefore, in this study, we aimed to compare the severity of negative symptoms in male smokers and nonsmokers before and after 12 weeks of treatment and to investigate whether smoking might affect drug efficacy.

## Materials and methods

### Subjects

One hundred and forty-five male patients with SCZ were recruited from Hebei Province Veterans hospital, Beijing Huilongguan Hospital and Wenzhou kangning hospital. Of all the 145 male patients, 60 were smokers and 85 were nonsmokers. Inclusion and exclusion criteria were described in a previous study^27^. Male first-episode inpatients, aged 16–45 years, diagnosed with SCZ by SCID-IV, were recruited for this study. Patients had a disease duration of no more than 5 years, cumulative duration of antipsychotic medication of no more than 2 weeks, and no significant medical comorbidities. The first episode was defined as a previous study^28^. Patients were excluded from this study if they had any other current categorical diagnosis of psychiatric disorders. All patients were treated with a flexible dose of antipsychotics for 12 weeks. In this 12-week observational study, patients were hospitalized and nurses monitored adherence to treatment.

The study protocol was approved by the Ethics Committee of Beijing Huilongguan Hospital, and written informed consent was obtained from all patients.

### Assessment

All patients were treated with flexible doses of oral antipsychotics for 12 weeks. The study consisted of three visits, conducted on day 1 (visit 1, screening), day 1 (visit 2, assessment of baseline), and at week 12 or after early discontinuation of the trial drug (visit 3, assessment of follow-up).

Six experienced psychiatrists assessed the severity of psychiatric symptoms using the Positive and Negative Syndrome Scale (PANSS)^29^. The PANSS scale was assessed at baseline and at week 12. The primary outcome was the change from baseline in the PANSS total score or subscores at week 12. After training, the repeated assessment showed that the inter-observer correlation coefficient was maintained at >0.8 for the PANSS total score.

### Statistical analysis

For patients who discontinued this clinical trial medication, the last observation carried forward (LOCF) was used. Analysis of variance (ANOVA) was performed to compare baseline demographic characteristics of the SCZ in smokers and nonsmokers. A repeated measures ANOVA was performed to analyze the different changes in PANSS total score and their subscale scores between smokers and non-smokers at baseline and the LOCF endpoint at week 12. Pearson correlation coefficients between symptom changes and demographic characteristics were calculated for smokers and nonsmokers, respectively. *p* values were adjusted using Bonferroni correction (B-adjusted *p*) as reported in previous studies^30–33^. Linear regression analyses were performed to identify factors associated with symptom improvement in SCZ patients. Regression analyses were performed to determine the relative contribution of smoking and other demographic variables (e.g., age, years of education, baseline BMI and onset age) to explain the variance in negative symptom improvement.

All data were analyzed with SPSS statistical analysis software (version 22.0; SPSS Inc., Chicago, IL), and significance thresholds were determined at *p* < 0.05.

## Results

At baseline, male smokers with SCZ were older, had a later age of onset and had a shorter education year than male nonsmokers with SCZ (all *p* < 0.05) (Table 1). Correlation analysis showed that clinical symptoms at baseline were associated with age, BMI, age at onset, and years of education (all *p* < 0.05). However, we did not find any differences in positive, negative symptoms, general psychopathology and PANSS total scores between smokers and nonsmokers in male patients with SCZ (*p* = 0.71, *p* = 0.11, *p* = 0.38, and *p* = 0.92).

**Table 1:** Demographic characteristics of smokers and nonsmokers with schizophrenia (SCZ).

Variable (mean ± SD)	Total sample (*n* = 145)	Nonsmokers (*n* = 85)	Smokers (*n* = 60)	*F*(*p*)
Age (years)	27.0 ± 8.2	25.5 ± 7.8	29.2 ± 8.3	7.2 (0.008)
Education (years)	10.1 ± 3.9	11.1 ± 3.8	8.7 ± 3.7	15.1 (<0.001)
BMI (kg/m^2^)	21.9 ± 3.4	21.7 ± 3.6	22.3 ± 3.0	0.9 (0.33)
Onset age (years)	25.6 ± 8.3	23.9 ± 7.4	27.6 ± 8.9	5.9 (0.02)

*BMI* body mass index, *SD* standard deviation.

After 12 weeks of treatment, the PANSS total score and each subscale score decreased significantly (all *p* < 0.01). The mean reduction in PANSS total score was 29.4 (95% CI 27.1–31.8) and the mean reductions in their subscale scores were 12.0 (95% CI 11.1–12.9) for positive symptoms, 4.9 (95% CI 4.2–5.6) for negative symptoms, and 12.7 (95% CI 11.5–13.9) for general psychopathology.

**Fig. 1:**
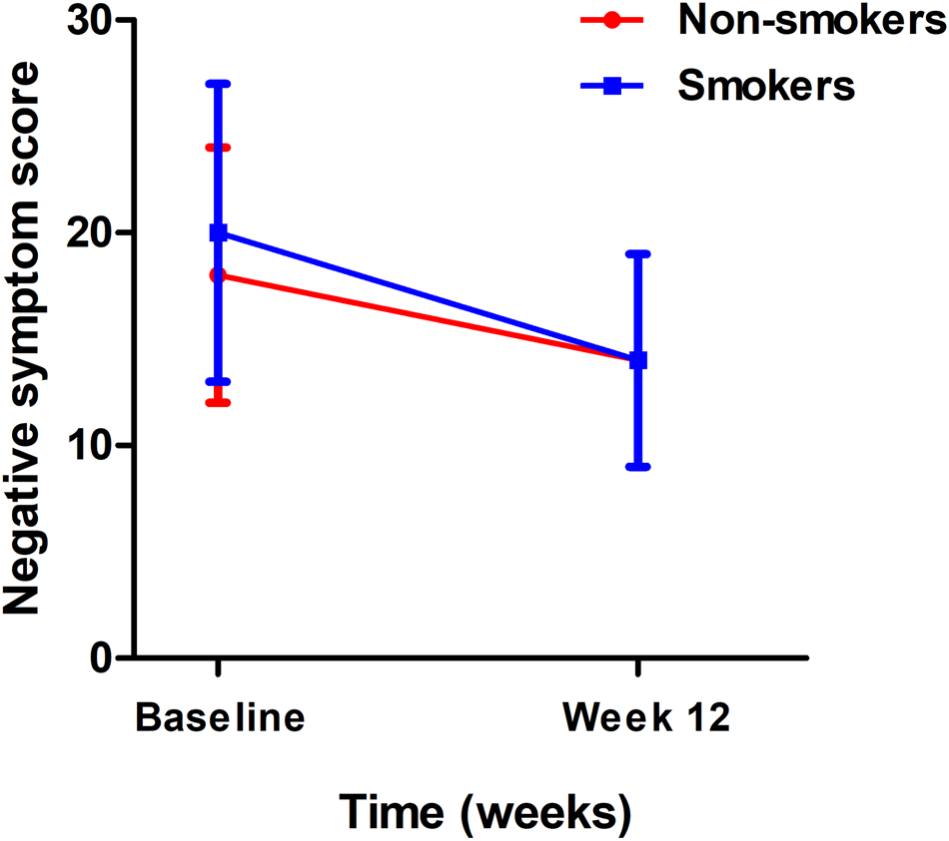
There is a significant in the improvements in negative symptoms between smokers and non-smokers.

In addition, we found significant differences in the improvement of negative symptoms, general psychopathology and total scores between smokers and nonsmokers with SCZ (all *p* < 0.05) (Table 2 and Fig. 1). After Bonferroni correction, only the improvements in negative symptoms and total score were significant between smoking and nonsmoking patients (all B-adjusted *p* < 0.05). The further paired *t-*test indicated that smokers showed greater improvement in negative symptoms than non-smokers [5.5 (95% CI 3.6–7.4) vs. 3.7 (95% CI 2.6–4.7), *p* < 0.05]. Additionally, the number of cigarettes was positively associated with negative symptom improvement (*r* = 0.57, *p* < 0.001).

**Table 2:** Comparisons of clinical symptoms before and after 12 weeks of risperidone monotherapy using the repeated measures ANOVA.

	Baseline		After treatment			
	Non-smokers	Smokers	Non-smokers	Smokers	Smoke *F*(*p*)	Interaction *F*(*p*)
Positive subscale	21.0 ± 5.8	22.4 ± 6.8	12.2 ± 4.9	12.2 ± 4.7	0.1 (0.79)	2.7 (0.10)
Negative subscale	17.8 ± 6.5	19.9 ± 8.0	14.7 ± 5.4	14.7 ± 6.4	1.0 (0.32)	7.3 (0.008)
General subscale	37.2 ± 10.4	36.7 ± 10.0	24.2 ± 6.0	24.9 ± 6.4	0.3 (0.60)	4.3 (0.04)
PANSS total score	78.7 ± 18.2	79.9 ± 19.2	49.5 ± 13.4	50.8 ± 13.2	0.8 (0.37)	6.4 (0.013)

*PANSS* the Positive and Negative Syndrome Scale.

Further regression analysis confirmed that smoking was a predictor of improvement in negative symptoms in male patients with SCZ (Beta = 0.26, *t* = 2.7, *p* = 0.008) (adjusted *R*^2^ = 0.15).

## Discussion

In this study, we found that male smokers with SCZ had greater clinical symptom improvement than male nonsmokers after 12 weeks of treatment, controlling for age, onset age, baseline BMI and education years.

Notably, in our study, negative symptoms of SCZ patients were slightly more severe at baseline. We hypothesize that the toxic component of cigarette plays a worsening role in clinical symptoms during the early and onset stages of SCZ, and therefore smokers per se have higher scores on negative symptom. A growing body of evidence suggests a common genetic predisposition and overlapping genetic roots for the development of smoking and SCZ^34,35^. The overlapping genetic risks combine with other risk factors for SCZ to exacerbate negative symptoms in smokers. However, the differences in negative symptoms between smokers and nonsmokers were not significant, although smokers had more than 2 points higher scores on negative symptoms than those of nonsmokers.

Consistent with our hypothesis, we found that after 12 weeks of treatment, male smokers with SCZ had more improvement in negative symptoms than male nonsmokers with SCZ. Many previous studies have focused on the relationship between smoking and the beneficial effects of antipsychotic medication on negative symptoms after treatment^36–39^. However, there is no consistent evidence that smoking has a beneficial effect on symptoms in SCZ^40^. In particular, there are few longitudinal and cohort studies on the relationship between smoking and clinical symptoms in DNFE patients with SCZ. Our prospective cohort study of first-episode drug naïve patients after a relatively short period (12 weeks) of treatment showed that smoking is associated with improvement in negative symptoms in male patients with SCZ. However, our results are inconsistent with previous studies, which reported that smoking was associated with more negative clinical outcomes^41^. These inconsistent results may be due to the type of antipsychotic, duration of treatment (12 weeks vs. 24 months), the severity of smoking dependence, and severity of psychotic symptoms at baseline.

Potential mechanisms for differential treatment response and symptom improvement between male smokers and non-smokers may be related to the interactive modulation of antipsychotics and smoking. The negative symptoms in SCZ may be the result of abnormalities in multiple neural networks (e.g., frontal-cortical-temporal, cortical-striatal; Millan et al.^42^). Current evidence supports the dopaminergic hypothesis as one of the pathophysiological hypotheses for negative symptoms in SCZ, which proposes that positive symptoms are the result of overactive dopaminergic neurotransmission in the limbic pathway, whereas negative symptoms are thought to be caused by low dopaminergic function in the frontal lobes and other limbic structures of the midbrain^7,43^. Nicotine is known to bind to nAChRs in the brain and alter neurotransmitter transmission^44,45^. Smoking can attenuate negative symptoms by reducing dopamine degradation and enhancing the effects of nicotine-mediated dopamine release in male SCZ patients, particularly in the inferior prefrontal cortex circuit^40,46–49^. Overall, our findings support a strong relationship between smoking and the improvement of negative symptoms after treatment with antipsychotics in male patients with first-episode SCZ. However, the number of smokers in SCZ patients was low (*n* = 60) due to the limited sample size and the low prevalence of smoking in first-episode SCZ patients. We also do not know whether the severity of dependence is associated with improvement in negative symptoms because we did not assess it with the Fagerstrom Test for Nicotine Dependence (FTND) in the present study. Therefore, the current study is preliminary and further longitudinal studies with large sample sizes are needed to investigate the role of smoking in the response of SCZ patients to treatment with various antipsychotic medications.

A previous study from our group investigated the association between smoking, oxidative stress and alleviation of clinical symptoms in patients with SCZ^50^, which demonstrated that smokers showed greater improvement in negative symptoms compared with nonsmokers. The present study recruited only male patients with SCZ and focused on the relationship between smoking and improvements in negative symptoms in SCZ after excluding the potential influence of gender on the findings.

There are some major limitations of the present study. First, due to the observational design of this study, we could not draw clear causal conclusions. Second, the smoking status collected by the self-report questionnaire used to measure smoking status may be a source of differential assessment error that may bias the association between smoking and negative symptoms. In addition, the dose of antipsychotics is flexible depending on the patient’s symptoms, and the daily dose of anitpsychotics used in each patient was not recorded in this study. We also did not measure the serum levels of antipsychotics. Therefore, we did not add the dose as a covariate. Third, the observation points after treatment were limited. Clinical symptoms were only assessed at two time points (baseline and week 12). In addition, the follow-up period is quite short (12 weeks). It would be better to extend this period and carry out a study with a longer period (minimum, 12 months) in the future. Fourth, we only examined the influence of smoking on the improvements in the severity of clinical symptoms. Further studies should extend the test to other broader tests such as the cognitive functions as assessed by MATRICS. Fifth, the number of non-smoking subjects is small compared to nonsmokers, which is different to real clinical practice, where the data indicate a higher prevalence of smokers. This may be due to the fact that all recruited patients were DNFE and were hospitalized during the 12 weeks of treatment.

In conclusion, our study showed that male smokers showed greater improvement in negative symptoms than male nonsmokers after 12 weeks of treatment with antipsychotics. Our findings provide further evidence for the relationship between smoking and negative symptoms in male patients with SCZ in the early stages of this disorder and after treatment with antipsychotics. However, it should be noted that the number of male smokers was small in this study. Further studies with a large sample size of male SCZ patients who smoke, with a longitudinal design of various antipsychotics, are needed to establish this relationship between smoking and the alleviation of negative symptoms in SCZ patients.
